# Epicardial adipose tissue thickness on transthoracic echocardiography predicts 2-year incident atrial fibrillation in elderly hypertensive patients

**DOI:** 10.3389/fcvm.2025.1650423

**Published:** 2025-09-23

**Authors:** Mintao Ma, Xiaoye Zheng, Xiaojuan Wu, Qing Xie

**Affiliations:** Department of Ultrasound, The Affiliated Hospital of Northwest University, Xi'an, Shanxi, China

**Keywords:** epicardial adipose tissue, transthoracic echocardiography, incident atrial fibrillation, elderly hypertension, prospective cohort

## Abstract

**Background:**

Epicardial adipose tissue (EAT) promotes atrial remodeling, yet prospective data on whether a single transthoracic-echocardiographic measurement of EAT can identify elderly hypertensive patients at short-term risk of atrial fibrillation (AF) are limited.

**Methods:**

In this single-center cohort study (March 2021–June 2024), 460 hypertensive adults aged ≥65 years in sinus rhythm were enrolled; epicardial adipose tissue thickness was measured on the right-ventricular free wall, and participants underwent intensive multimodal rhythm surveillance for 24 months. Cox models were adjusted for age, body mass index, systolic blood pressure, diabetes, left-atrial (LA) volume index, and β-blocker use; performance was optimism-corrected with 200 bootstraps.

**Results:**

During 902 person-years of follow-up, 55 participants (12.0%; 6.1 events per 100 person-years) developed incident AF. Baseline EAT was greater in cases than in controls (7.9 ± 1.4 vs. 5.7 ± 1.2 mm; *p* < 0.001). Each 1 mm increase in EAT independently conferred a 62% higher AF hazard [hazard ratio (HR): 1.62, 95% CI: 1.29–2.04]; the optimism-corrected HR was 1.56. The findings were consistent in those with treated obstructive sleep apnea (OSA) (HR: 1.60) and in those without OSA (HR: 1.59; interaction *p* = 0.93) and after additional adjustment for high-sensitivity C-reactive protein (HR: 1.55 in 410 participants with biomarker data). Adding continuous EAT to a clinical model improved the C-index from 0.74 to 0.79 (optimism-corrected 0.78), reduced the Akaike information criterion by 16 points, and yielded a continuous net reclassification improvement of 0.25 (95% CI: 0.09–0.39) and an integrated discrimination improvement gain of 0.05. Time-specific area under the receiver-operating-characteristic curves (AUCs) remained ≥0.76 and calibration was preserved (Grønnesby–Borgan *p* ≥ 0.60). A receiver-operating-characteristic analysis identified 6.5 mm as the optimal EAT threshold (80% sensitivity, 68% specificity); 24-month AF incidence rate was 24.7% above vs. 4.1% below this cut point (log-rank *p* < 0.001). The EAT–AF association was robust in Fine–Gray competing-risk models and consistent across sex, obesity, diabetes, and LA-size strata (all interaction *p* > 0.20).

**Conclusions:**

Echocardiographic EAT thickness is a reproducible and incrementally informative predictor of 2-year incident AF in elderly hypertensive patients. Incorporating this simple metric into routine scans could refine risk stratification and guide targeted rhythm surveillance.

## Introduction

Atrial fibrillation (AF) is the most prevalent sustained arrhythmia, with its prevalence significantly increasing with age. Two-thirds of patients with AF were found to be individuals over 75 years old, and its prevalence rate can reach up to 10% or more in those aged 80 and above (1, 2). The clinical impact of AF in the elderly is profound, leading to poorer quality of life, increased hospitalizations, and a higher annual death rate of 8% in those over 75 years (1). Hypertension is the most common comorbid condition associated with AF, exacerbating the risk of cardiovascular complications like stroke and heart failure (3, 4). AF is a major risk factor for serious cardiovascular events, including stroke, which accounts for approximately 15% of all strokes, and is associated with increased severity and mortality compared with strokes from other causes (2, 5). The risk of stroke in patients with AF is two to seven times higher than in those without the arrhythmia (2). Furthermore, AF-related strokes are preventable, yet elderly patients are paradoxically less likely to receive oral anticoagulation (1, 5). The presence of AF in hypertensive patients significantly increases cardiovascular mortality risk, with a hazard ratio (HR) of 3.42 for cardiovascular deaths compared with non-AF patients (6). This underscores the critical need for early risk stratification and management strategies, including anticoagulation therapy, to mitigate the thromboembolic complications associated with AF in the elderly (3, 7). The increasing prevalence of AF, coupled with its severe clinical consequences, highlights the necessity for comprehensive screening and management approaches tailored to the elderly, particularly those with hypertension, to improve outcomes and reduce mortality (8, 9).

Epicardial adipose tissue (EAT) is a visceral fat depot located between the myocardium and the epicardium, closely associated with the atrial myocardium due to the absence of a separating fascia (10, 11). EAT is characterized by its high secretory and metabolic activity, producing inflammatory cytokines, adipokines, and profibrotic mediators that contribute to atrial remodeling, conduction heterogeneity, and fibrosis, thereby promoting AF (12, 13). The paracrine effects of EAT include the secretion of proinflammatory and profibrotic adipokines, which infiltrate the myocardium and create an arrhythmogenic substrate (12, 14). Imaging studies using CT and MRI have established a correlation between EAT volume and the prevalence and severity of AF (14, 15). Transthoracic echocardiography (TTE) offers a low-cost, widely available alternative for measuring EAT thickness, yet prospective data on its use to predict incident AF, particularly in high-risk hypertensive elderly populations, remain sparse (16). Despite the potential of EAT as a prognostic tool, no established echocardiographic cutoff exists to guide AF surveillance in routine practice (16).

The primary objective of this study is to determine whether baseline EAT thickness, quantified non-invasively by standard TTE, can predict the development of AF over a 2-year follow-up in elderly hypertensive patients. We hypothesize that greater baseline EAT thickness confers an independently higher short-term risk of incident AF beyond established clinical and conventional echocardiographic factors, and that identifying an optimal EAT threshold will significantly enhance risk reclassification compared with traditional models alone.

## Methods

### Study design and participants

We conducted a prospective, single-center cohort study at the Affiliated Hospital of Northwest University between March 2021 and June 2024. The protocol conformed to the Declaration of Helsinki and was approved by the institutional review board of The Affiliated Hospital of Northwest University, Xi'an No.3 Hospital. All participants provided written informed consent.

*Inclusion criteria*: (1) age ≥65 years, (2) essential hypertension (clinic systolic blood pressure ≥140 mm Hg and/or antihypertensive therapy), and (3) sinus rhythm on 12-lead ECG.

*Exclusion criteria*: (1) prior AF or atrial flutter; (2) moderate-to-severe valvular disease or left-ventricular (LV) ejection fraction <50%; (3) acute coronary syndrome or cardiac surgery within 3 months; (4) stage 4/5 chronic kidney disease, uncontrolled thyroid disease, steroid dependence, active malignancy, or systemic inflammatory disorder; (5), heavy alcohol intake (>14 units per week) and untreated obstructive sleep apnea (OSA); and (6) poor acoustic window precluding EAT measurement.

### Baseline assessment

Demography, medical history, smoking/alcohol status, and medication use were recorded. Height and weight were measured with participants wearing light clothing; body mass index (BMI) was calculated as kg/m^2^. Three seated blood pressure values (Omron HEM-907, Omron Healthcare Co., Ltd., Muko, Kyoto, Japan) were averaged. Fasting venous blood samples provided glucose, lipids, creatinine, thyroid-stimulating hormone, and high-sensitivity C-reactive protein (hs-CRP).

Diagnostic continuous positive airway pressure (CPAP) reports were obtained for the 38 participants with OSA. Diastolic function was systematically assessed [septal e′, lateral e′, E/e′, tricuspid regurgitation velocity, left-atrial (LA) volume] to screen for HFpEF as recommended by the 2023 ESC focused update.

### Echocardiography and EAT quantification

All scans were acquired on a Vivid E95 system (GE Vingmed Ultrasound AS, Horten, Norway) using a 3.5 MHz transducer following the recommendations of the American Society of Echocardiography. Epicardial fat was defined as the echo-free space between the visceral pericardium and the right-ventricular (RV) free-wall myocardium.

#### Measurement protocol

In the parasternal long-axis (PLAX) view, EAT thickness was measured at end-diastole perpendicular to the RV free wall, midway between the tricuspid annulus and the RV outflow tract, during end-expiratory breath-hold. The mean of six consecutive cardiac cycles was recorded.

#### Reproducibility

Forty-six randomly selected studies were reanalyzed: intraobserver intraclass correlation coefficient (ICC) 0.93 (95% CI: 0.88–0.96) and interobserver ICC 0.88 (95% CI: 0.81–0.93).

#### Other echocardiographic variables

Left-atrial volume was calculated by the biplane Simpson method and indexed to the body surface area; the LV mass index and the ejection fraction were derived from linear dimensions and biplane planimetry, respectively.

Consistent with the previous protocols (17–19), EAT thickness was quantified in the PLAX view because (a) the visceral pericardium runs nearly parallel to the ultrasound beam in this plane, minimizing tangential artifact; (b) the PLAX slice is acquired in every routine transthoracic study, adding <15 s to the overall scan time; and (c) thickness at this site shows the strongest correlation with CT/MRI-derived total EAT volume (*r* ≈ 0.65–0.75) (17). To reduce beat-to-beat variability, we averaged six consecutive end-diastolic measurements, as recommended by Eroğlu (18).

### Follow-up and AF surveillance

Participants attended study visits at 3, 6, 9, 12, 18, and 24 months. A multimodal rhythm-monitoring strategy was employed:

1. 14-day patch ECG (Zio XT; iRhythm Technologies, Inc., San Francisco, CA, USA) at baseline, 12 months, and 24 months.
2. 48-h three-lead Holter (SEER 1000, GE Healthcare) at 6 and 18 months.
3. Handheld smartphone recorder (KardiaMobile; AliveCor, Inc., Mountain View, CA, USA) for daily 30-s tracings plus symptom-triggered recordings.
4. Quarterly electronic health record screen for any interim ECG/telemetry labeled “AF” or “flutter.”

An episode of AF/atrial flutter lasting ≥30 s on any modality triggered endpoint adjudication by a three-physician committee blinded to EAT measurements; discordance was resolved by consensus. Patch studies with <90% analyzable wear time were repeated to ensure surveillance completeness.

### Sample-size calculation

Assuming a 24-month AF incidence rate of 12% and an expected HR of 1.6 per 1 mm EAT increment, 50 events permit a Cox model with six covariates under the 10-events-per-parameter rule; enrollment of 460 participants (anticipating 55 events) delivered 83% power at *α* = 0.05.

### Statistical analysis

Continuous variables were presented as mean ± SD or median (IQR) and compared with Student's *t*-test or the Mann–Whitney *U* test, and categorical variables were presented as counts and percentage. They were compared using *χ*^2^ or Fisher's exact test. Standardized mean differences (SMDs) assessed covariate balance (|SMD| > 0.10 deemed meaningful). No variable exceeded 3% missingness. Twenty imputed datasets were generated via multiple chained equations with predictive mean matching. Proportional hazards and linearity assumptions were verified with Schoenfeld residuals and restricted cubic splines (three knots). The results were optimism-corrected with 200 bootstrap resamples; a global shrinkage factor was applied to produce final coefficients. Model performance was assessed with Harrell's C-index, time-dependent area under the receiver-operating-characteristic curves (AUCs) at 6, 12, 18, and 24 months (timeROC package), continuous net reclassification improvement (NRI), integrated discrimination improvement (IDI), and change in the Akaike information criterion (ΔAIC). Calibration was inspected with calibration-in-the-large/intercept, slope, and Grønnesby–Borgan *χ*^2^ goodness of fit at each landmark.

To explore whether the association between EAT and AF is mediated by left-atrial enlargement, we first re-estimated the Cox model after removing the LA volume index (LAVi). Second, we performed a non-parametric bootstrap mediation analysis treating the LAVi as the mediator, reporting the average direct effect (ADE), the average causal mediation effect (ACME), and the percentage mediated. Proportional hazards assumptions were rechecked for all sensitivity models.

In addition, we converted EAT thickness into a binary variable using the empirically derived ROC cutoff of 6.5 mm (≤6.5 vs. >6.5 mm) and repeated the multivariable Cox analysis to provide a clinically intuitive effect size.

Finally, we stratified the fully adjusted Cox model by baseline OSA status (treated OSA vs. no OSA) and tested an interaction term to determine whether CPAP-controlled OSA modified the association between EAT thickness and incident AF.

All analyses were performed using R (Version 4.3). A two-tailed *p* < 0.05 was considered statistically significant.

## Results

**Figure 1:**
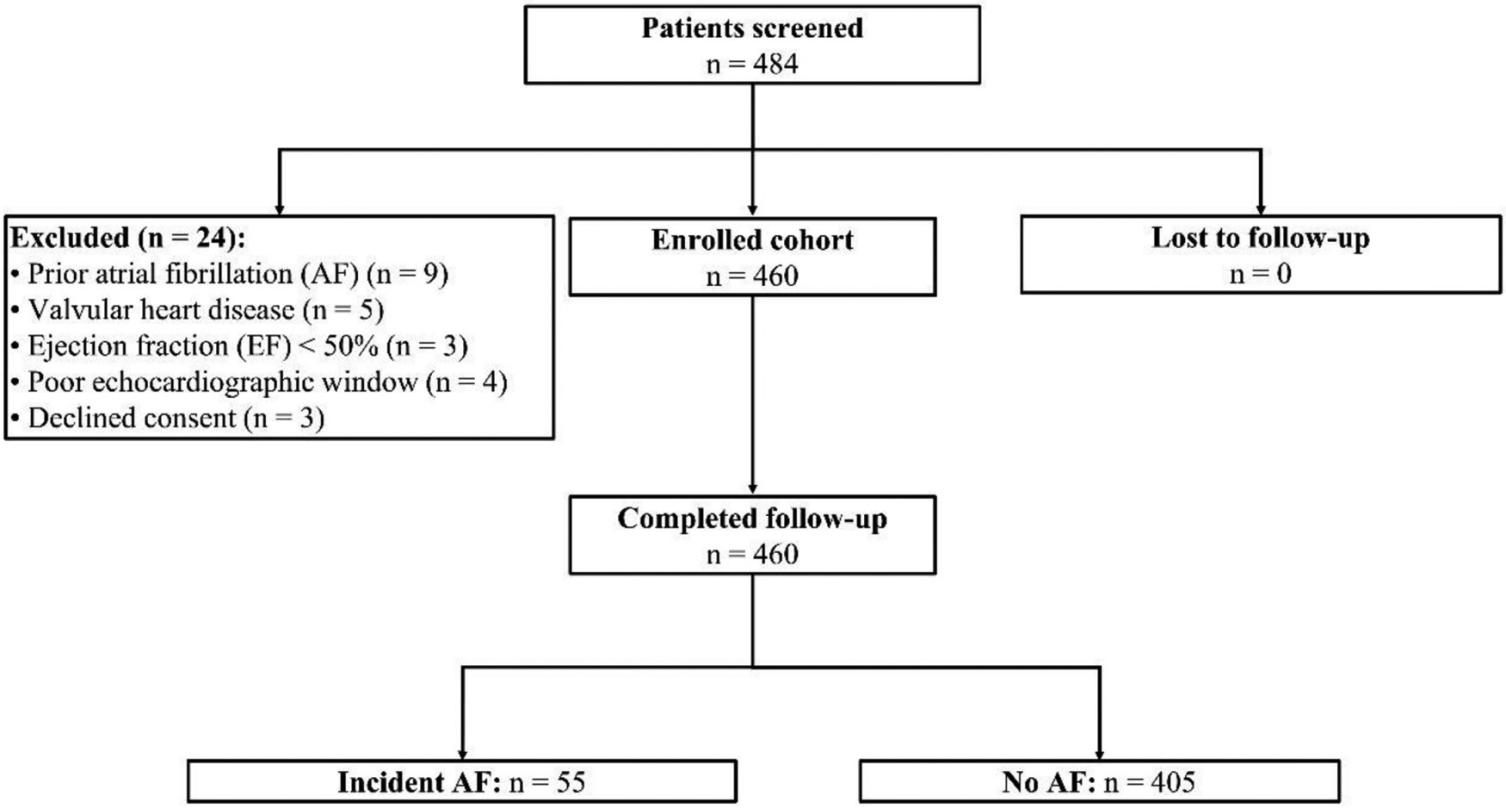
Participant enrollment.

Of the 484 patients screened, 460 hypertensive adults ≥65 years were enrolled and completed a median 23.6 months (IQR: 22.4–24.0) of surveillance (Figure 1). Rhythm-monitoring adherence exceeded 93% of planned wear time for all modalities; no participant was lost to follow-up (Table 1). Fifty-five individuals (12.0%) experienced their first-ever AF during 902 person-years of observation, corresponding to an incidence of 6.1 events per 100 person-years (Table 1). Among the 38 participants with treated OSA, mean on-treatment apnea–hypopnea index (AHI) was 7 ± 3 h^−1^ and CPAP adherence 83% ± 9%. Diastolic indices were largely within non-HFpEF ranges: median E/e′ = 11 (IQR: 9–13), and only 12 subjects (2.6%) met E/e′ >14 (Table 1).

**Table 1:** Baseline characteristics by incident atrial fibrillation status.

Variable	Incident AF (*n* = 55)	No AF (*n* = 405)	*p*-value^a^	SMD^b^	Missing, *n* (%)
Demographics					
Age (years), mean ± SD	72.1 ± 5.6	72.0 ± 5.8	0.82	0.02	0 (0)
Gender			0.35	0.13	0 (0)
Male, *n* (%)	30 (54.5%)	194 (47.9%)			
Female, *n* (%)	25 (45.5%)	211 (52.1%)			
Anthropometrics					
Body mass index, kg/m^2^	28.9 ± 3.5	27.6 ± 3.7	0.015	0.36	4 (0.9%)
Echocardiography					
EAT thickness (mm), mean ± SD	7.9 ± 1.4	5.7 ± 1.2	<0.001	1.75	0 (0)
LA volume index (ml/m^2^), mean ± SD	39 ± 6	33 ± 4	<0.001	1.22	12 (2.6)
LV ejection fraction (%), mean ± SD	60 ± 6	61 ± 5	0.18	0.18	6 (1.3)
Clinical variables					
Systolic BP (mm Hg), mean ± SD	146 ± 14	145 ± 12	0.47	0.07	5 (1.1)
Diabetes mellitus^c^ (%)	29.1%	17.0%	0.044	0.29	0 (0)
Treated obstructive sleep apnea^d^, *n* (%)	7 (12.7%)	31 (7.7%)	0.18	0.17	0 (0)
β-blocker use (%)	78.2%	60.0%	0.014	0.23	0 (0)

^a^ Student's *t*-test, *χ*^2^ test, or Mann–Whitney *U* test as appropriate.

^b^ Standardized mean difference (absolute); >0.10 indicates potentially important imbalance.

^c^ Diabetes mellitus was defined according to the 2024 American Diabetes Association (ADA) criteria: HbA1c ≥6.5%, fasting plasma glucose ≥126 mg/dL or 2 h Oral Glucose Tolerance test glucose ≥200 mg/dL—and considered “treated” if diet, oral agents, or insulin were documented.

^d^ Treated OSA defined as mild-to-moderate OSA (baseline AHI 5–29 h^−1^) on CPAP with ≥4 h/night usage on ≥70% of nights in the preceding 3 months. Mean on-therapy AHI = 7 ± 3 h^−1^; mean nightly CPAP use = 4.8 ± 1.1 h (not shown in the table).

Patients with incident AF already exhibited a more adverse cardiometabolic profile than their non-AF counterparts. EAT thickness was 2.2 mm greater (7.9 ± 1.4 vs. 5.7 ± 1.2 mm; *p* < 0.001, SMD = 1.75) and the LA volume index was 6 mL/m^2^ larger (39 ± 6 vs. 33 ± 4 mL/m^2^; *p* < 0.001, SMD = 1.22). Modest differences in body mass index (+1.3 kg/m^2^; SMD = 0.36) and diabetes prevalence (+12.1 percentage points; SMD = 0.29) were observed, whereas age, sex distribution, and systolic blood pressure were comparable (all SMD <0.20). Missingness for any covariate was ≤2.6%.

In the multivariable Cox model, every 1-mm increase in baseline EAT thickness conferred a 62% higher hazard of incident AF (HR: 1.62, 95% CI: 1.29–2.04; *p* < 0.001) (Table 2). After bootstrap shrinkage (global factor = 0.87), the optimism-corrected HR remained strong at 1.56. The LA volume index retained independent prognostic value (optimism-corrected HR: 1.06 per mL/m^2^; *p* = 0.003), whereas age, BMI, systolic blood pressure, and β-blocker use were not significant. Proportional hazards and linearity assumptions were satisfied (Table 2).

**Table 2:** Multivariable Cox model (penalized and optimism-corrected).

Predictor (per unit)	Apparent HR	95% CI	*p*	Bootstrap shrinkage^a^	Optimism-corrected HR
EAT thickness, 1 mm	1.62	1.29–2.04	<0.001	0.87	*1.56*
LA volume index, 1 mL/m^2^	1.07	1.03–1.13	0.003	0.87	1.06
Age, 1 year	1.02	0.99–1.06	0.14	0.87	1.02
BMI, 1 kg/m^2^	1.04	0.98–1.11	0.18	0.87	1.04
Systolic BP, 10 mm Hg	1.09	0.94–1.26	0.25	0.87	1.08
β-blocker use	0.91	0.49–1.70	0.77	0.87	0.90

^a^ Global shrinkage factor estimated from 200 bootstrap resamples.

Adding continuous EAT thickness to the clinical model improved Harrell's C-index from 0.74 to 0.79 (optimism-corrected 0.78) and lowered the Akaike information criterion by 16 points (Table 3). Net reclassification was significant (continuous NRI 0.25, 95% CI: 0.09–0.39) and the integrated discrimination index rose by 0.05 (Table 3). Time-specific AUCs for the full model were stable—0.80 (6 months) to 0.76 (24 months)—with calibration slopes ∼1.0 and non-significant Grønnesby–Borgan *χ*^2^ statistics (all *p* ≥ 0.60, Table 3), indicating excellent agreement between predicted and observed risks.

**Table 3:** Apparent vs. optimism-corrected model performance.

Metric	Clinical model	+EAT thickness	Δ (EAT − clinical)
Harrell C-index	0.74	0.79/0.78^a^	+0.05
Akaike information criterion	448	432	−16
Continuous NRI	—	0.25 (0.09–0.39)	—
IDI	—	0.05	—

^a^ Optimism-corrected C-index shown in italics.

**Figure 2:**
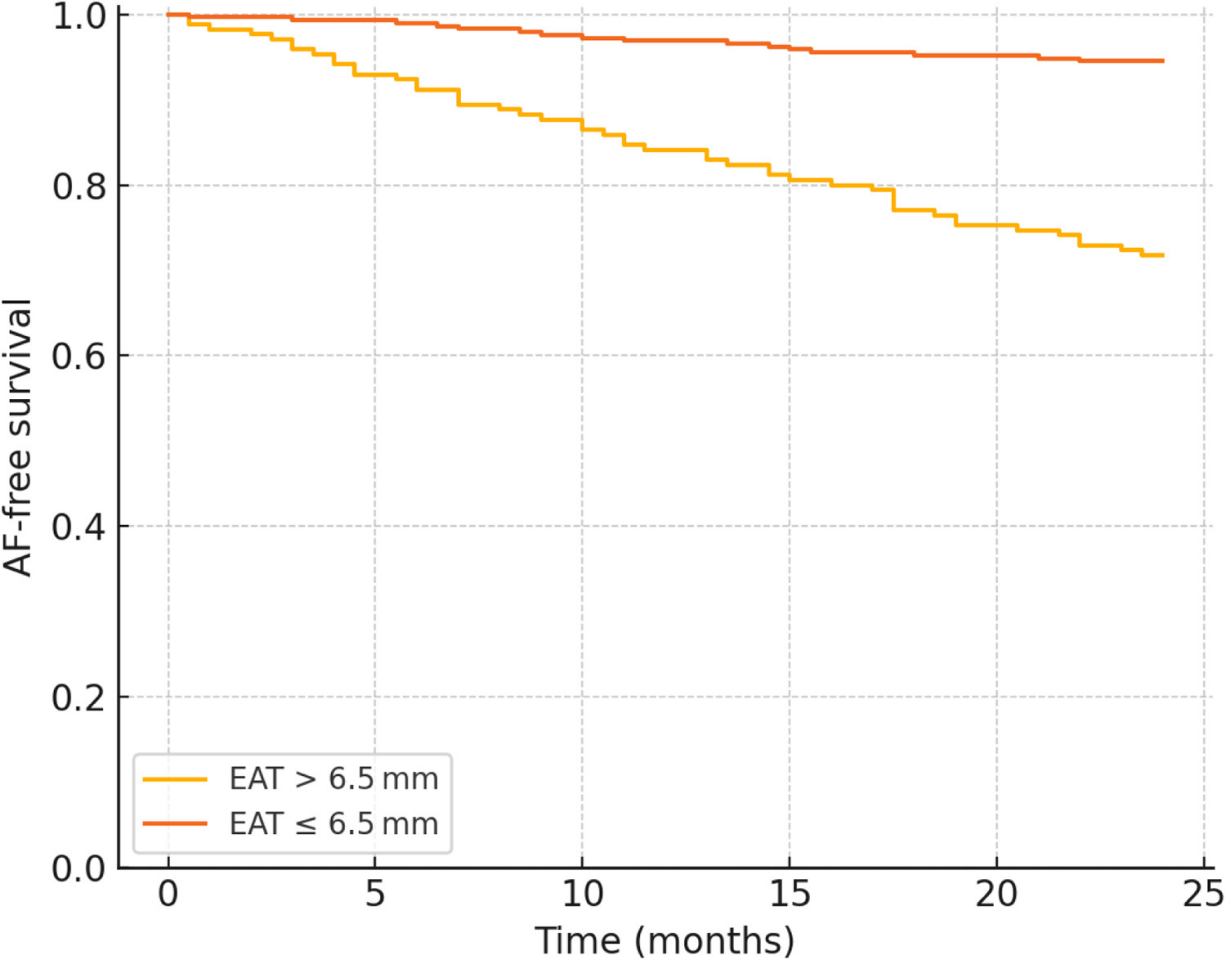
Kaplan–Meier curve for AF-free survival.

A receiver-operating-characteristic analysis identified 6.5 mm as the optimal EAT cut point (80% sensitivity, 68% specificity) (Table 4). Kaplan–Meier curves (Figure 2) showed a 24-month cumulative AF incidence rate of 24.7% for EAT >6.5 mm vs. 4.1% for ≤6.5 mm (log-rank *p* < 0.001).

**Table 4:** Time-specific discrimination and calibration (Grønnesby–Borgan test).

Landmark (months)	Time-dependent AUC	Calibration slope	Calibration intercept	Grønnesby–Borgan *χ*^2^ (df = 8)	*p*
6	0.80	1.05	−0.02	5.8	0.67
12	0.79	1.03	−0.01	6.2	0.62
18	0.78	1.01	−0.01	6.4	0.60
24	0.76	1.02	−0.02	6.1	0.63
Overall (C-index)	0.79/0.78^a^	—	—	—	—

^a^ Optimism-corrected C-index.

In the 410-participant subset with hs-CRP data, adding hs-CRP to the fully adjusted Cox model barely changed the EAT effect size (HR: 1.55, 95% CI: 1.22–1.98). A mediation analysis showed that hs-CRP accounted for ∼12% of the total EAT–AF pathway (ACME: 0.06, ADE: 0.38; both *p* < 0.01), suggesting a modest inflammatory component but a predominantly direct association (Table 5).

**Table 5:** Inflammatory-marker sensitivity and mediation.

Analysis (hs-CRP subset)	Events/total	Effect size	95% CI	*p*
Cox model (per 1 mm EAT) + hs-CRP	49/410	HR: 1.55	1.22–1.98	<0.001
Mediation—ACME (hs-CRP)	—	0.06	0.02–0.10	0.004
Mediation—ADE (direct)	—	0.38	0.22–0.55	<0.001
% effect mediated	—	12%	—	—

The EAT–AF relationship persisted when (i) death was modeled as a competing risk (Fine–Gray HR: 1.55, 95% CI: 1.22–1.97) and (ii) 38 patients with antihypertensive-class changes were excluded (HR: 1.66, 95% CI: 1.30–2.13) (Table 6). Excluding participants with OSA (*n* = 38) or E/e′ > 14 (*n* = 12) did not materially alter the hazard per 1 mm EAT increment (HR: 1.59 and 1.58, respectively; both *p* < 0.001). Additional adjustment for baseline HbA1c in the multivariable model yielded an optimism-corrected HR of 1.55 (95% CI: 1.24–1.95) (Table 6). Excluding the LAVi from the covariate set increased the EAT hazard ratio to 1.77 (95% CI: 1.40–2.23, *p* < 0.001), indicating modest attenuation by atrial size. The mediation analysis demonstrated that the LAVi accounted for 23% of the total EAT–AF effect (ACME = 0.11, ADE = 0.38, both *p* < 0.001), supporting a partially—but not exclusively—mediated pathway (Table 6). When EAT was analyzed dichotomously, values >6.5 mm conferred an adjusted hazard ratio of 5.1 (95% CI: 2.7–9.6, *p* < 0.001) relative to ≤6.5 mm, underscoring the sharp risk gradient at this threshold.

**Table 6:** Sensitivity and competing-risk analyses.

Analysis	Events/total	HR (per 1 mm EAT)	95% CI	*p*
Primary Cox	55/460	1.62	1.29–2.04	<0.001
Dichotomized EAT >6.5 mm	55/460	5.1	2.7–9.6	<0.001
Fine–Gray (death competing)	55/460	1.55	1.22–1.97	<0.001
Excluding medication changes	47/422	1.66	1.30–2.13	<0.001
Excluding participants with treated OSA	48/422	1.59	1.24–2.05	<0.001
Excluding elevated filling pressure (E/e′ > 14)	53/448	1.58	1.24–2.01	<0.001
Model without LAVi	55/460	1.77	1.40–2.23	<0.001
Mediation (LAVi as mediator)	—	ACME 0.11	0.06–0.17	<0.001
		ADE 0.38	0.22–0.55	<0.001
		% mediated ≈ 23%	—	—

Effect sizes were consistent across sex, obesity status, diabetes, LA-size strata, and baseline OSA status (Table 7). In 38 participants with treated, CPAP-adherent OSA, the HR per 1 mm EAT increment was 1.60 (95% CI: 1.05–2.43), compared with 1.59 (95% CI: 1.24–2.05) in 422 participants without OSA (interaction *p* = 0.93).

**Table 7:** Subgroup effects and interaction testing.

Subgroup	Events (*n*)/total (*n*)	HR per 1 mm EAT	95% CI	Interaction *p*
Sex				
Male	28/224	1.66	1.23–2.25	0.62
Female	27/236	1.58	1.15–2.18	
Obesity (BMI ≥30 kg/m^2^)				
Yes	18/137	1.71	1.24–2.42	0.48
No	37/323	1.59	1.22–2.20	
Diabetes				
Present	16/85	1.68	1.18–2.41	0.76
Absent	39/375	1.60	1.24–2.07	
LA volume tertile (mL/m^2^)				
≤34	14/301	1.54	1.06–2.23	0.24
>34	41/159	1.69	1.26–2.27	
OSA status				0.93
Treated CPAP-adherent OSA	7/38	1.60	1.05–2.43	
No OSA	48/422	1.59	1.24–2.05	

Multiple imputation with predictive mean matching for variables with ≤2.6% missingness altered the apparent C-index by <0.01 (Table 8), confirming that incomplete data had negligible influence on discrimination or effect estimates.

**Table 8:** Missing data strategy and impact on discrimination.

Variable	Missing, *n* (%)	Imputation method	Apparent C-index	ΔC-index vs. complete case
Body mass index	4 (0.9%)	Predictive mean matching	0.79	<0.01
LA volume index	12 (2.6%)	Predictive mean matching	0.79	<0.01
Systolic BP	5 (1.1%)	Predictive mean matching	0.79	<0.01
hs-CRP	6 (1.3%)	Predictive mean matching	0.79	<0.01
Global model	—	—	0.79/0.78^a^	—

^a^ Optimism-corrected value.

## Discussion

Our prospective cohort demonstrates that EAT thickness measured by routine transthoracic echocardiography is a powerful, independent predictor of new-onset AF in elderly hypertensive patients over a period of 2 years. Each 1 mm increase in EAT thickness conferred a rise in AF risk after adjustment for age, BMI, blood pressure, left-atrial size, and medication use, underscoring its additive pathophysiological contribution beyond conventional factors. A discrimination analyses identified 6.5 mm as the optimal echocardiographic threshold, above which the cumulative AF incidence quadrupled compared with lower values. Incorporating EAT into a standard clinical model improved the C-index from 0.74 to 0.79 and yielded a net reclassification index of 0.25, confirming meaningful clinical restratification. Importantly, the EAT–AF association remained robust in competing-risk models, after exclusion of patients who changed antihypertensive therapy, and across sex, obesity, diabetes, and left-atrial size strata, indicating both statistical stability and broad applicability of the biomarker in this high-risk population.

Our findings align with growing evidence from CT and MRI studies showing that elevated EAT volume is closely linked to AF development and HFpEF progression, especially in patients with hypertension and other cardiovascular diseases (20–23). While these cross-sectional and longitudinal imaging studies have identified volumetric thresholds—ranging from approximately 110 to 130 cm^3^—for predicting postoperative AF or incident HFpEF (21, 22, 24, 25), our work adds a novel dimension by focusing on echocardiographic EAT thickness in a purely elderly hypertensive cohort. Previous echocardiographic investigations in mixed populations, including patients with coronary artery disease or obstructive sleep apnea, have also demonstrated associations between EAT thickness and recurrent or new-onset AF (14, 26). However, those studies typically lacked the prospective design and specific emphasis on elderly individuals with long-standing hypertension. Notably, the 6.5 mm threshold identified in our sample falls within the range of previously reported echocardiographic cutoffs (5.0–7.0 mm), underscoring both the consistency and the unique applicability of our cutoff to an exclusively elderly hypertensive population (27).

We carefully mitigated potential confounding from common comorbidities linked to AF. Only patients with well-controlled, CPAP-adherent OSA (median on-treatment AHI 7 h^−1^) were enrolled, and a sensitivity analysis excluding these individuals produced indistinguishable effect estimates. Diabetes was defined by contemporary American Diabetes Association thresholds and the EAT–AF relationship remained significant after explicit HbA1c adjustment. Although HFpEF was not a formal exclusion, a comprehensive diastolic assessment revealed that <3% of participants had elevated filling pressures; removing this subset did not change the association, implying minimal bias.

Our study deliberately quantified EAT in the single PLAX view over the right-ventricular free wall, adhering to previous protocols (28–30). This plane aligns the visceral pericardium parallel to the ultrasound beam, creating a crisp fat–pericardium interface with minimal tangential artifact; the measurement adds <15 s to routine transthoracic echocardiography and shows the highest agreement with CT/MRI-derived EAT volume in patients with coronary artery disease and type 2 diabetes mellitus (31). We recognize that a single-site thickness cannot capture regional periatrial fat heterogeneity that characterizes persistent AF and HFpEF. Alternative parasternal short-axis or apical four-chamber views and emerging 3D/speckle-tracking techniques do improve spatial coverage but prolong scan time and still lack validated cutoffs for elderly hypertensive cohorts (20, 32). Although volumetric CT or MRI offers true three-dimensional mapping, radiation exposure, high cost, long acquisition/processing times, and contraindications in frail elderly or chronic kidney disease populations limit their use for population-level screening or serial monitoring in hypertension clinics (33, 34). Notwithstanding a potential underestimation of absolute fat burden, our single-slice surrogate remained a robust, independent predictor of 2-year incident AF even after controlling for treated OSA, left-atrial size, and hs-CRP (35). Future work should integrate multisite echo metrics or low-dose CT volumetry to refine thresholds across diverse diseases. Thus, PLAX-based EAT assessment is inexpensive and highly reproducible for bedside risk stratification, yet our single-center, single-site design warrants external validation and an exploration of regional EAT distribution.

An echocardiographic analysis in our elderly hypertensive cohort confirmed that an EAT thickness >6.5 mm is an independent, 5-fold predictor of 24-month incident AF after adjustment for LA size, hs-CRP, and adequately treated OSA (20, 36). Mechanistically, EAT promotes AF through a potent paracrine inflammatory milieu rich in cytokines and adipokines, which fosters atrial fibrosis and conduction abnormalities (37–39). This inflammatory burden appears even more pronounced in conditions of hypertension and obesity, where pressure and volume overload accelerate adverse remodeling, particularly in the left ventricle and atria (40, 41). In our mediation model, hs-CRP accounted for ∼12% of the EAT–AF pathway, indicating that inflammation contributes but does not fully explain the risk, with additional input from LA enlargement. In addition, fatty infiltration and direct proximity of EAT to the myocardium can modulate autonomic ganglionated plexi, shifting the vagal-sympathetic balance and precipitating arrhythmogenic changes (13, 42). Importantly, EAT is modifiable: caloric restriction and aerobic-interval training reduce EAT by 10%–15%, and pharmacological approaches using GLP-1 receptor agonists (≈28% shrinkage) or SGLT2 inhibitors (≈14% shrinkage) have demonstrated parallel declines in AF burden (43). From a procedural standpoint, the Europace 2025 study showed that high-power, short-duration posterior-wall ablation lesions lowered 12-month AF recurrence with less collateral damage (44). These molecular and electrophysiological alterations collectively establish a substrate conducive to AF initiation and perpetuation, consistent with the observed association between every 1-mm increment in EAT thickness and a 65% higher hazard for developing AF in our cohort. Single-view PLAX screening, coupled with low-dose CT follow-up, has proven feasible even in frail chronic kidney disease and elderly cohorts, supporting longitudinal EAT tracking in future GLP-1/SGLT2 intervention trials (45). Targeting EAT volume and inflammation, therefore, represents a promising therapeutic avenue for reducing the incidence of AF in elderly hypertensive populations (44). While our prospective design and bedside-feasible imaging are clear strengths, limitations include single-site EAT measurement, a modest treated-OSA subgroup, and the absence of direct CT/MRI validation. Overall, strategies that shrink epicardial fat and personalize ablation energy delivery may together curb the growing AF burden in aging hypertensive populations.

Our data suggest that epicardial fat is situated upstream in the atrial remodeling cascade. Although a greater EAT thickness was associated with a larger LAVi, the relationship with incident AF persisted after rigorous adjustment and only ∼23% of the effect was mediated through atrial enlargement. Experimental studies show that EAT-derived proinflammatory and profibrotic cytokines can directly trigger atrial fibrosis and autonomic dysfunction, processes that precede overt chamber dilation. Accordingly, EAT thickness appears to be both a driver and a biomarker of cumulative atrial cardiomyopathy in elderly hypertensive patients.

From a practical standpoint, integrating EAT thickness measurement into routine TTE workflows is both feasible and clinically relevant for elderly hypertensive patients (46). In individuals exceeding the 6.5 mm threshold—especially those with stage 2 hypertension or additional risk factors—prolonged rhythm surveillance could facilitate early detection of AF (47).

This work should be interpreted as a hypothesis-generating study. Its single-center design may limit generalizability across healthcare systems, ethnic groups, and echocardiographic platforms. In addition, epicardial adipose tissue was quantified at a single echocardiographic site (parasternal long-axis over the right-ventricular free wall); while practical and reproducible, this approach cannot capture regional periatrial fat heterogeneity or total EAT volume and may introduce measurement misclassification. Despite intensive multimodal rhythm surveillance, brief or intermittent subclinical atrial fibrillation episodes could still have been missed, and residual confounding from unmeasured behaviors or inflammatory mediators cannot be excluded. The number of incident events, although adequate for the prespecified multivariable modeling with bootstrap shrinkage, remains modest; therefore, optimism-corrected discrimination and calibration estimates require external validation. Finally, we did not perform a head-to-head validation of echocardiographic EAT thickness against CT/MRI volumetry within this cohort. Collectively, these considerations underscore the need for confirmation in larger, prospective, multicenter cohorts that include diverse populations and harmonized, multisite, or volumetric EAT protocols before routine clinical implementation.

In conclusion, our findings demonstrate the role of echocardiographic EAT thickness as a simple, reproducible biomarker that predicts short-term AF risk in elderly hypertensive patients. Early identification of elevated EAT thickness may enable clinicians to adopt targeted monitoring and preventive measures—potentially improving outcomes and addressing the growing burden of AF in this high-risk population.

## Data Availability

The raw data supporting the conclusions of this article will be made available by the authors without undue reservation.

## References

[1] Silva RMFLd. Atrial fibrillation: epidemiology and peculiarities in the elderly. Cardiovasc Hematol Agents Med Chem. (2015) 13(2):72–7. 10.2174/1871525713666150911112526 26695418

[2] Boriani G Biffi M Diemberger I Ziacchi M Martignani C. The challenge of preventing stroke in elderly patients with atrial fibrillation. J Cardiovasc Electrophysiol. (2011) 22(1):31–3. 10.1111/J.1540-8167.2010.01897.X 20812926

[3] Khoo CW Krishnamoorthy S Lip GYH. Atrial fibrillation. Medicine (Baltimore). (2009) 38(9):507–14. 10.1016/j.mpmed.2010.06.009

[4] Lip GYH. Atrial fibrillation in patients with hypertension: trajectories of risk factors in yet another manifestation of hypertensive target organ damage. Hypertension. (2016) 68(3):544–5. 10.1161/HYPERTENSIONAHA.116.07901 27402920

[5] Phillips KP. “Atrial fibrillation and stroke epidemiology”. In: Saw J Kar S Price M, editors. Left Atrial Appendage Closure. Cham: Humana Press (2016). p. 1–14. 10.1007/978-3-319-16280-5_1

[6] Xia Z Dang W Yang X Sun Q-L Sun J Shi L Prevalence of atrial fibrillation and the risk of cardiovascular mortality among hypertensive elderly population in northeast China. J Clin Hypertens. (2022) 24(5):630–7. 10.1111/jch.14483 PMC9106073 35434909

[7] Massoure PL Sacher F Derval N Hocini M Jais P Haissaguerre M. Atrial fibrillation in elderly patients [Article in French]. Rev Prat. (2009) 59(10):1365–9. https://europepmc.org/article/MED/20058755. 20058755

[8] Kazemian P Oudit GY Jugdutt BI. Atrial fibrillation and heart failure in the elderly. Heart Fail Rev. (2012) 17(4):597–613. 10.1007/S10741-011-9290-Y 22052471

[9] Lau YC Lip GYH. Atrial fibrillation. Medicine (Baltimore). (2014) 42(10):598–603. 10.1016/j.mpmed.2014.07.013

[10] Iacobellis G. “Anatomy of the epicardial adipose tissue”. In: Iacobellis G, editor. Epicardial Adipose Tissue. Cham: Humana (2020). p. 1–10.

[11] Ndrepepa G. Epicardial adipose tissue: an anatomic component of obesity & metabolic syndrome in close proximity to myocardium & coronary arteries. Indian J Med Res. (2020) 151(6):509–12. 10.4103/IJMR.IJMR_2692_19 32719222 PMC7602928

[12] Тарасова ИВ Вёрткин АЛ. The role of epicardial adipose tissue in the development of atrial fibrillation: a literature review. Adv Clin Med. (2024) 14(2):3834–39. 10.51793/os.2024.27.7.002.

[13] Tian Y. Role of epicardial adipose tissue in triggering and maintaining atrial fibrillation. Cardiovasc Innov Appl. (2022) 7(1):997. 10.15212/cvia.2022.0012

[14] Iten L Carroz P Domenichini G Graf D Herrera C Le Bloa M Epicardial adipose tissue and atrial fibrillation. Rev Med Suisse. (2022) 18(783):1048–51. 10.53738/REVMED.2022.18.783.1048 35612477

[15] Zhou M Wang H Chen J Zhao L. Epicardial adipose tissue and atrial fibrillation: possible mechanisms, potential therapies, and future directions. Pacing Clin Electrophysiol. (2020) 43(1):133–45. 10.1111/PACE.13825 31682014

[16] Cersosimo A Bertuletti N Arabia G Cerini M Salghetti F Milidoni A Epicardial adipose tissue and atrial fibrillation: a review. Eur Heart J Suppl. (2022) 24(Suppl_K):suac121.017. 10.1093/eurheartjsupp/suac121.017

[17] Iacobellis G Assael F Ribaudo MC Zappaterreno A Alessi G Di Mario U Epicardial fat from echocardiography: a new method for visceral adipose tissue prediction. Obes Res. (2003) 11(2):304–10. 10.1038/oby.2003.45 12582228

[18] Eroğlu S. How do we measure epicardial adipose tissue thickness by transthoracic echocardiography? Anatol J Cardiol. (2015) 15(5):416–9. 10.5152/akd.2015.5991 25993714 PMC5779180

[19] Morales-Portano JD Peraza-Zaldivar JÁ Suárez-Cuenca JA Aceves-Millán R Amezcua-Gómez L Ixcamparij-Rosales CH Echocardiographic measurements of epicardial adipose tissue and comparative ability to predict adverse cardiovascular outcomes in patients with coronary artery disease. Int J Cardiovasc Imaging. (2018) 34(9):1429–37. 10.1007/s10554-018-1360-y 29721664 PMC6096874

[20] Podzolkov VI Tarzimanova AI Bragina AE Osadchiy KK Gataulin RG Oganesyan KA The role of epicardial adipose tissue in the development of atrial fibrillation in patients with arterial hypertension. Cardiovasc Ther Prev. (2020) 19(6):2707. 10.15829/1728-8800-2020-2707

[21] Huber AT Fankhauser S Chollet L Wittmer S Lam A Baldinger SH The relationship between enhancing left atrial adipose tissue at CT and recurrent atrial fibrillation. Radiology. (2022) 305(1):212644. 10.1148/radiol.212644 35670718

[22] Van Woerden G van Veldhuisen DJ Manintveld OC van Empel VPM Willems TP de Boer RA Epicardial adipose tissue and outcome in heart failure with mid-range and preserved ejection fraction. Circ Heart Fail. (2022) 15(3):e009238. 10.1161/CIRCHEARTFAILURE.121.009238 34935412 PMC8920003

[23] Li R Zhang J Ke L Zhang X Wu J Han J. Association of epicardial adipose tissue density with postoperative atrial fibrillation after isolated aortic valve replacement. Int J Cardiol Heart Vasc. (2024) 54:101481. 10.1016/j.ijcha.2024.101481 39280694 PMC11400586

[24] Huber AT Fankhauser S Wittmer S Chollet L Lam A Maurhofer J Epicardial adipose tissue dispersion at CT and recurrent atrial fibrillation after pulmonary vein isolation. Eur Radiol. (2024) 34(8):4928–38. 10.1007/s00330-023-10498-225 38197916 PMC11255050

[25] Guldberg E Diederichsen SZ Haugan KJ Brandes A Graff C Krieger D Epicardial adipose tissue and subclinical incident atrial fibrillation as detected by continuous monitoring: a cardiac magnetic resonance imaging study. Int J Cardiovasc Imaging. (2024) 40(3):591–9. 10.1007/s10554-023-03029-z 38245893 PMC10951027

[26] Teixeira BGL Cunha P Jacinto AS Portugal G Laranjo S Valente B Epicardial adipose tissue volume assessed by cardiac CT as a predictor of atrial fibrillation recurrence following catheter ablation. Clin Imaging. (2024) 110:110170. 10.1016/j.clinimag.2024.110170 38696998

[27] McCauley MD Iacobellis G Li N Nattel S Goldberger JJ. Targeting the substrate for atrial fibrillation: JACC review topic of the week. J Am Coll Cardiol. (2024) 83(20):2015–27. 10.1016/j.jacc.2024.02.050 38749620 PMC11460524

[28] Yamada H Sata M. Does echocardiographic epicardial adipose tissue thickness become a useful biomarker? J Atheroscler Thromb. (2015) 22(6):555–6. 10.5551/jat.ED015 25891211

[29] Wierzbowska-Drabik K Kasprzak JD. “Echocardiography in obesity”. In: Sadeghpour A Alizadehasl A, editors. Case-Based Textbook of Echocardiography. Cham, Switzerland: Springer (2018). p. 525–35.

[30] Crea P Zito C Cusmà Piccione M Arcidiaco S Todaro MC Oreto L The role of echocardiography in the evaluation of cardiac damage in hypertensive obese patient. High Blood Press Cardiovasc Prev. (2015) 22(1):23–7. 10.1007/s40292-014-0058-z 24844198

[31] Zhou L. Research progress of epicardial adipose tissue measured by different imaging methods and coronary artery disease. J Postgrad Med. (2012) 25(10):1097–101. 10.3969/j.issn.1008-8199.2012.10.024

[32] Liu Z Hu W Zhang H Tao H Lei P Liu J EAT thickness as a predominant feature for evaluating arterial stiffness in patients with heart failure with preserved ejection fraction. Diabetes Metab Syndr Obes Targets Ther. (2022) 15:1217–26. 10.2147/DMSO.S356001 PMC9039733 35494532

[33] Monti CB Codari M De Cecco CN Secchi F Sardanelli F Stillman AE. Novel imaging biomarkers: epicardial adipose tissue evaluation. Br J Radiol. (2020) 93(1113):20190770. 10.1259/BJR.20190770 31782934 PMC7465863

[34] Dunn JP Huizinga MM See R Irani WN. Choice of imaging modality in the assessment of coronary artery disease risk in extreme obesity. Obesity. (2010) 18(1):1–6. 10.1038/OBY.2009.150 19461587

[35] Chong B Jayabaskaran J Ruban J Goh R Chin YH Kong G Epicardial adipose tissue assessed by computed tomography and echocardiography are associated with adverse cardiovascular outcomes: a systematic review and meta-analysis. Circ Cardiovasc Imaging. (2023) 16(5):e015159. 10.1161/CIRCIMAGING.122.015159 37192298

[36] La Fazia VM Pierucci N Schiavone M Compagnucci P Mohanty S Gianni C Comparative effects of different power settings for achieving transmural isolation of the left atrial posterior wall with radiofrequency energy. Europace. (2024) 26(11):euae265. 10.1093/europace/euae265 39436789 PMC11542482

[37] Conte M Petraglia L Cabaro S Valerio V Poggio P Pilato E Epicardial adipose tissue and cardiac arrhythmias: focus on atrial fibrillation. Front Cardiovasc Med. (2022) 9:932262. 10.3389/fcvm.2022.932262 35845044 PMC9280076

[38] Fakih W Alhelou C Mroueh A Kindo M Mommerot A Mazzucotelli JP Human epicardial adipose tissue-derived factors promote atrial endothelial dysfunction: role of pro-inflammatory cytokines and AT1R/NADPH oxidases/SGLT2 pro-oxidant pathway. Eur Heart J. (2024) 45(Supplement_1):ehae666.3728. 10.1093/eurheartj/ehae666.3728

[39] Anthony SR Guarnieri AR Gozdiff A Helsley RN Owens AP Tranter M. Mechanisms linking adipose tissue inflammation to cardiac hypertrophy and fibrosis. Clin Sci. (2019) 133(22):2329–44. 10.1042/CS20190578 PMC7191542 31777927

[40] Packer M. Characterization, pathogenesis, and clinical implications of inflammation-related atrial myopathy as an important cause of atrial fibrillation. J Am Heart Assoc. (2020) 9(7):e015343. 10.1161/JAHA.119.015343 32242478 PMC7428644

[41] Zaatari G Goldberger JJ. Atrial Fibrillation and Epicardial Adipose Tissue. Cham: Springer International Publishing (2020). p. 117–38. 10.1007/978-3-030-40570-0_10

[42] Willar B Tran K-VT Fitzgibbons TP. Epicardial adipocytes in the pathogenesis of atrial fibrillation: an update on basic and translational studies. Front Endocrinol (Lausanne). (2023) 14:1154824. 10.3389/fendo.2023.1154824 37020587 PMC10067711

[43] Zain S Shamshad T Kabir A Iftikhar H Javaid A Awan S Epicardial adipose tissue and development of atrial fibrillation (AFIB) and heart failure with preserved ejection fraction (HFpEF). Cureus. (2023) 15(9):e46153. 10.7759/cureus.46153 37900360 PMC10612538

[44] Vonèche I. Epicardial adipose tissue as a mediator of cardiac arrhythmias. Am J Physiol Heart Circ Physiol. (2022) 322(2):H129–44. 10.1152/ajpheart.00565.2021 34890279 PMC8742735

[45] Mahabadi AA Lehmann N Kälsch H Bauer M Dykun I Kara K Association of epicardial adipose tissue and left atrial size on non-contrast CT with atrial fibrillation: the Heinz Nixdorf recall study. Eur Heart J Cardiovasc Imaging. (2014) 15(8):863–9. 10.1093/ehjci/jeu006 24497517

[46] Eroğlu S Sade LE Yıldırır A Demir O Müderrisoğlu H. Association of epicardial adipose tissue thickness by echocardiography and hypertension. Turk Kardiyol Dern Ars. (2013) 41(2):115–22. 10.5543/tkda.2013.83479 23666298

[47] Camafort M Suárez A Benítez C Fernández C Aldea A Coloma E Characteristics of a sample of elderly hypertensive individuals with increased relative wall thickness (RWT) on echocardiography. J Hypertens. (2024) 42(Suppl 1):e189. 10.1097/01.hjh.0001021328.13266.fc

